# Supplementary material for: AMF-SporeChip provides new insights into arbuscular mycorrhizal fungal asymbiotic hyphal growth dynamics at the cellular level
Source: Lab Chip. 2024 Feb 16;24(7):1930–46. doi: 10.1039/d3lc00859b (PMC10964749; doi:10.1039/d3lc00859b)
Supplement: LC-024-D3LC00859B-s001 [file LC-024-D3LC00859B-s001.pdf]

Electronic Supplementary Material (ESI) for Lab on a Chip.
This journal is © The Royal Society of Chemistry 2024

Description of Additional Supplementary Files

AMF-SporeChip provides new insights into arbuscular mycorrhizal fungal asymbiotic hyphal growth dynamics at the cellular level.

Felix Richter[1], Maryline Calonne-Salmon[2], Marcel G. A. van der Heijden[3,4,5], Stéphane Declerck[2] and Claire E. Stanley[1]*

1 Department of Bioengineering, Imperial College London, London, SW7 2AZ, United Kingdom

2 Laboratory of Mycology, Earth and Life Institute, Université catholique de Louvain, B-1348 Louvain-la-Neuve, Belgium

3 Agroecology and Environment Research Division, Agroscope, 8046 Zurich, Switzerland

4 Department of Evolutionary Biology and Environmental Studies, University of Zurich, 8057 Zurich, Switzerland

5 Institute of Environmental Biology, Utrecht University, 3584 CS Utrecht, The Netherlands

* Correspondence: claire.stanley@imperial.ac.uk
+44 (0)20 75942893
www.imperial.ac.uk/claire.stanley

File name: Supplementary Movie 1

Description: Germination of *Rhizophagus irregularis* MUCL 41833 within the *AMF-SporeChip*. A time-lapse experiment was recorded over 19 hours and 15 minutes with 15 min intervals between image acquisitions using phase contrast microscopy. The movie shows the germination of one spore of *R. irregularis* MUCL 41833 in a microchannel with a single, straight hypha emerging from the germination site. Scale bar = 100 µm. Time stamp format = hh:mm:ss.

File name: Supplementary Movie 2

Description: Germination of *Rhizophagus irregularis* MUCL 43194 within the *AMF-SporeChip*. A time-lapse experiment was recorded over 55 hours with 20 min intervals between image acquisitions using phase contrast microscopy. The movie shows the germination of several spores of *R. irregularis* MUCL 43194 in a microchannel with multiple, curly hyphae emerging from each germination site. In some spores, prior to germination, a change in phase contrast down the subtending hypha can be observed, indicating a directed discharge of cellular contents. Scale bar = 50 µm. Time stamp format = hh:mm:ss.

File name: Supplementary Movie 3

Description: Germination of *Rhizophagus intraradices* MUCL 49410 within the *AMF-SporeChip*. A time-lapse experiment was recorded over 78 hours with 20 min intervals between image acquisitions using phase contrast microscopy. The movie shows the germination of one spore of *R. intraradices* MUCL 49410 in a microchannel with a single hypha emerging from the germination site, which branches several times, shortly behind the initial point of germination. Scale bar = 100 µm. Time stamp format = hh:mm:ss.

File name: Supplementary Movie 4

Description: Spores of *Rhizophagus intraradices* MUCL 49410 internally collapsing within the *AMF-SporeChip*. A time-lapse experiment was recorded over 23 hours and 20 minutes with 20 min intervals between image acquisitions using phase contrast microscopy. The movie shows spores of *R. intraradices* MUCL 49410 collapsing internally, and suddenly ejecting storage vesicles and cellular content into the attached hyphae. Scale bar = 100 µm. Time stamp format = hh:mm:ss.

File name: Supplementary Movie 5

Description: Spore forming in asymbiotic hypha of *Rhizophagus irregularis* MUCL 43194. A time-lapse experiment was recorded over 30 hours with 20 min intervals between image acquisitions using phase contrast microscopy. The movie shows a hypha of *R. irregularis* MUCL 43194 forming a spore starting with bulging in a centre position in the hypha. Scale bar = 100 µm. Time stamp format = hh:mm:ss.

File name: Supplementary Movie 6

Description: Hypha of *Gigaspora margarita BEG 34* growing into the *AMF-SporeChip*. A time-lapse experiment was recorded over 25 hours and 20 minutes with 20 min intervals between image acquisitions using phase contrast microscopy. The movie shows a hypha of *Gi. margarita* BEG 34 growing into a dead-end microchannel. Hyphal branching, cytoplasmic retraction and septa formation can be observed. Scale bar = 150 µm. Time stamp format = hh:mm:ss.

File name: Supplementary Movie 7

Description: Tip-to-tip anastomosis between two hyphae of *Rhizophagus irregularis* MUCL 43194 within the *AMF-SporeChip*. A time-lapse experiment was recorded over 21 hours with 20 min intervals between image acquisitions using phase contrast microscopy. The movie shows two hyphae of *R. irregularis* MUCL 43194 approaching each other in a directed, stop-and-go manner, prior to tip-to-tip anastomosis. Scale bar = 100 µm. Time stamp format = hh:mm:ss.

File name: Supplementary Movie 8

Description: Tip-to-tip anastomosis between a hypha and a germination site of *Rhizophagus irregularis* MUCL 43194 within the *AMF-SporeChip*. A time-lapse experiment was recorded over 83 hours and 40 minutes with 20 min intervals between image acquisitions using phase contrast microscopy. The movie shows a hypha of *R. irregularis* MUCL 43194 approaching a germination site of a subtending hypha, to which it anastomoses. After 57 h, a change in phase contrast shifting from the subtending hypha into the approaching hypha can be observed, suggesting a transfer of cellular contents through the newly formed connection, followed by the formation of new branches. Scale bar = 50 µm. Time stamp format = hh:mm:ss.

File name: Supplementary Movie 9

Description: Tip-to-side anastomosis between two hyphae of *Rhizophagus irregularis* MUCL 43194 within the *AMF-SporeChip*. A time-lapse experiment was recorded over 20 hours and 20 minutes with 20 min intervals between image acquisitions using phase contrast microscopy. The movie shows a hypha of *R. irregularis* MUCL 43194 approaching a second hypha and anastomoses with it from the side. After successful merging, fluctuation of cellular contents between both hyphae can be observed. Scale bar = 25 µm. Time stamp format = hh:mm:ss.

File name: Supplementary Movie 10

Description: Dynamic hyphal reactions of *Rhizophagus irregularis* MUCL 41833 within the *AMF-SporeChip*. A time-lapse experiment was recorded over 45 hours and 45 minutes with 15 min intervals between image acquisitions using phase microscopy. The movie shows the growth of a hypha of *R. irregularis* MUCL 41833 being obstructed by an obstacle, causing a change in growth behaviour, involving growth arrest, cytoplasmic retraction, directional

changes, lateral branching as well as reversal of cytoplasmic retraction. Scale bar = 100 µm. Time stamp format = hh:mm:ss.

File name: Supplementary Movie 11

Description: Dynamic hyphal behaviour of *Rhizophagus irregularis* MUCL 41833 within the *AMF-SporeChip*. A time-lapse experiment was recorded over 92 hours and 30 minutes with 15 min intervals between image acquisitions using phase microscopy. The movie shows the highly dynamic growth of a hypha of *R. irregularis* MUCL 41833 involving growth arrest, irreversible cytoplasmic retraction with obstacle contact as well as in open space, complete and incomplete reversible cytoplasmic retraction, directional changes and lateral branching. Scale bar = 100 µm. Time stamp format = hh:mm:ss.

File name: Supplementary Movie 12

Description: "Hit and split" event of *Rhizophagus irregularis* MUCL 43194 within the *AMF-SporeChip*. A time-lapse experiment was recorded over 20 hours and 20 minutes with 20 min intervals between image acquisitions using phase microscopy. The movie shows frontal collision of a hypha of *R. irregularis* MUCL 43194 with an obstacle, causing apical branching in opposite directions, called "hit and split". The hypha grows slightly underneath the obstacle, caused by an imperfect bonding of the structure. Scale bar = 100 µm. Time stamp format = hh:mm:ss.

File name: Supplementary Movie 13

Description: Growth arrest of *Rhizophagus irregularis* MUCL 43194 hypha within the *AMF-SporeChip*. A time-lapse experiment was recorded over 99 hours and 40 minutes with 20 min intervals between image acquisitions using phase microscopy. The movie shows the growth of a hypha of *R. irregularis* MUCL 43194 arresting and remaining in a "frozen" immobile state without cytoplasmic retraction for 70 h before finally retracting cytoplasm. Scale bar = 100 µm. Time stamp format = hh:mm:ss.

File name: Supplementary Movie 14

Description: Dynamic hyphal reaction of *Rhizophagus irregularis* MUCL 43194 within the *AMF-SporeChip*. A time-lapse experiment was recorded over 20 hours and 20 minutes with 20 min intervals between image acquisitions using phase microscopy. The movie shows a hypha of *R. irregularis* MUCL 43194 colliding with an obstacle. The hypha keeps growing thus bending and pushing itself away from the obstacle. Scale bar = 50 µm. Time stamp format = hh:mm:ss.
